# Accelerating electrostatic particle-in-cell simulation: A novel FPGA-based approach for efficient plasma investigations

**DOI:** 10.1371/journal.pone.0302578

**Published:** 2024-06-03

**Authors:** Abedalmuhdi Almomany, Muhammed Sutcu, Babul Salam K. S. M. Kader Ibrahim

**Affiliations:** 1 Department of Electrical & Computer Engineering, Gulf University for Science & Technology, Hawally, Kuwait; 2 Department of Engineering Management, Gulf University for Science & Technology, Hawally, Kuwait; 3 Department of Computer Engineering, Hijjawi Faculty for Engineering Technology, Yarmouk University, Irbid, Jordan; UFCSPA: Universidade Federal de Ciencias da Saude de Porto Alegre, BRAZIL

## Abstract

Particle-in-cell (PIC) simulation serves as a widely employed method for investigating plasma, a prevalent state of matter in the universe. This simulation approach is instrumental in exploring characteristics such as particle acceleration by turbulence and fluid, as well as delving into the properties of plasma at both the kinetic scale and macroscopic processes. However, the simulation itself imposes a significant computational burden. This research proposes a novel implementation approach to address the computationally intensive phase of the electrostatic PIC simulation, specifically the Particle-to-Interpolation phase. This is achieved by utilizing a high-speed Field Programmable Gate Array (FPGA) computation platform. The suggested approach incorporates various optimization techniques and diminishes memory access latency by leveraging the flexibility and performance attributes of the Intel FPGA device. The results obtained from our study highlight the effectiveness of the proposed design, showcasing the capability to execute hundreds of functional operations in each clock cycle. This stands in contrast to the limited operations performed in a general-purpose single-core computation platform (CPU). The suggested hardware approach is also scalable and can be deployed on more advanced FPGAs with higher capabilities, resulting in a significant improvement in performance.

## Introduction

Plasma stands as the predominant state of matter in the universe, constituting over 99% of the visible cosmos [1]. It represents the fourth state of matter, characterized as an ionized gas encompassing both negatively charged electrons and positively charged ions, whose positions are influenced by magnetic and electrical fields. The particles within plasma interact not only with each other but also with the surrounding electromagnetic fields in space. Understanding these intricate interactions and their evolution is crucial, necessitating thorough modeling and simulation [2]. Simulation of plasma involves the characterization and description of its state. Various models can be employed in these simulations, including single-particle [3], kinetic, fluid, hybrid kinetic-fluid, gyrokinetic, and a system of many particles [4]. Investigating processes at the kinetic scale is essential for identifying plasma properties such as particle acceleration induced by turbulence and fields [5]. Fluid models prove valuable in studying macroscopic processes and properties of dense and cold (collisional) plasma, where Maxwell’s equations must be solved. Kinetic treatment, on the other hand, is employed to explore microscopic processes like particle acceleration, magnetic reconnection, and turbulence, addressing kinetic scale effects and their contributions to the macroscopic picture [6].

The Particle-In-Cell (PIC) method emerges as a particularly intuitive and straightforward approach for plasma simulation [7]. In essence, PIC simulation simplifies the common N-body problem, where all particles interact with each other due to Coulomb collision. PIC simulations are often applied to sparse-density plasmas, where particle collisions can be disregarded, resulting in collision-less simulations. In PIC simulations, particles are influenced by a collective electromagnetic field generated by both the particles themselves and any externally applied boundary conditions [8]. This approach significantly reduces the computational complexity from *O(N*^*2*^*)* to *O(NlogN)*, with *N* being the number of particles. PIC simulation proves to be one of the most suitable and promising techniques for studying macroscopic effects, enabling the exploration of phenomena beyond the scope of fluid models, such as particle acceleration and distribution through interactions with self-consistently generated electromagnetic or electrostatic fields [2, 9]. Through PIC simulations, we can analyze the physical properties of the system kinetically, obtaining information on position, velocity, and electric fields at each grid point. This allows for a comprehensive investigation into the microscopic properties of the system, considering the abundance of particles [10, 11].

**Fig 1:**
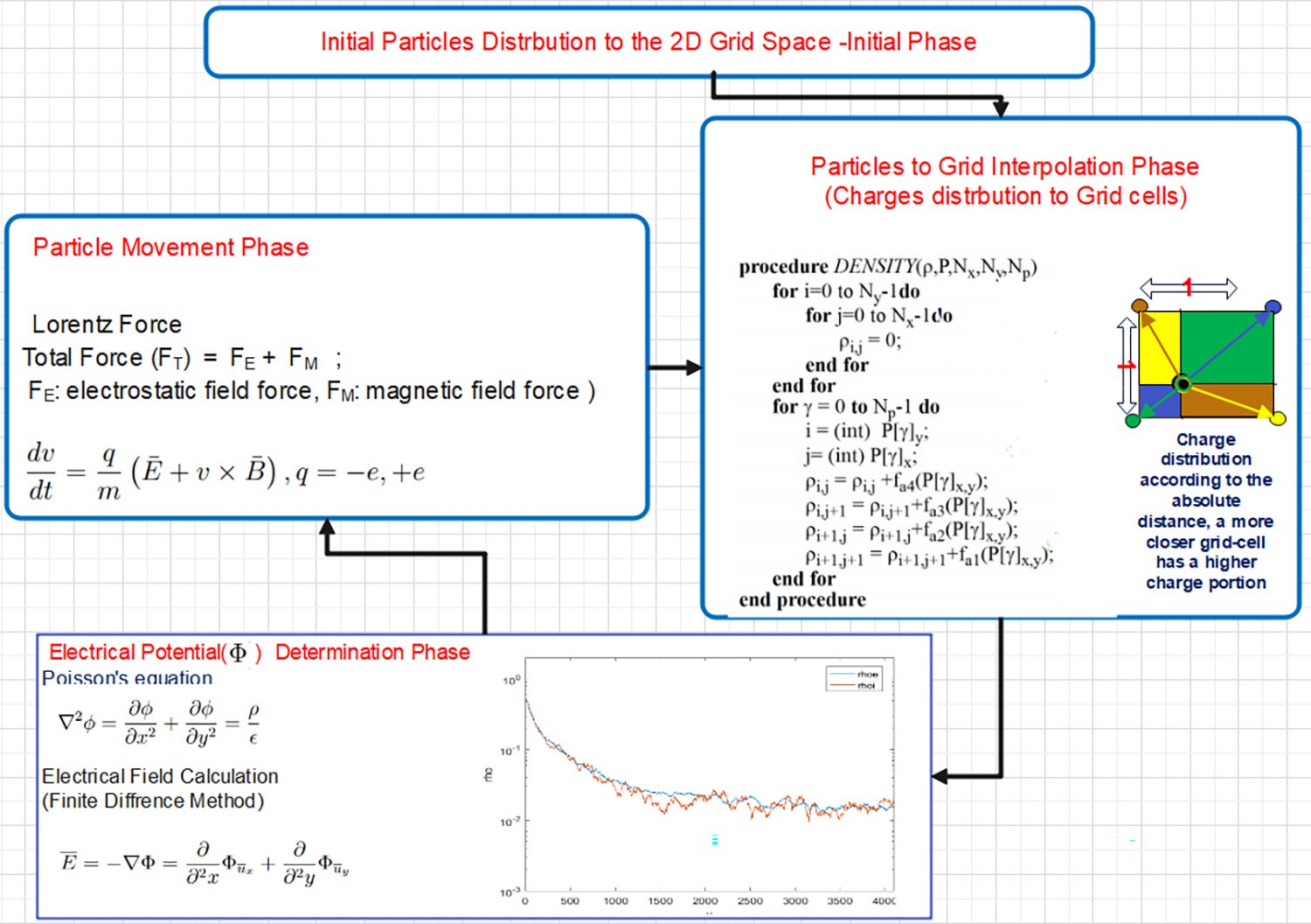
PIC simulation phases.

The proposed simulation employs a grid size of *32x32* cells, with *ΔX* representing the distance between neighboring cells along the X-dimension and *ΔY* along the Y-dimension. Both *ΔX* and *ΔY* are maintained at small values, less than the Debye length characteristics of plasma, ensuring a more accurate modeling of particle interactions. Both *ΔX* and *ΔY* are normalized to one for consistency [12]. The underlying assumption is that particles are randomly distributed within the grid area. Fig 1 illustrates the primary loop of the PIC simulation, highlighting its key functionalities.

Following the uniform distribution of particles in the 2D grid space, the Grid Interpolation Phase is initiated. In this simulation code, the proposed function confines the impact of each particle’s electric charge to the four surrounding grid points. Following that, the computation of the Electric-Field component vector, denoted as E, takes place for each grid point in the Grid Space Field Calculation Phase. The resultant field vector, covering the four grid points within a cell, induces acceleration for each particle within that specific cell. As part of the boundary conditions, the simulation considers a magnetic field that is non-uniform yet time-invariant. Particles within the simulation exhibit the flexibility to move freely within the designated region or exit through either the top or bottom. Furthermore, each iteration in the simulation corresponds to a predetermined and fixed simulation time step. Executing various plasma simulation models, including Particle-in-Cell (PIC) simulations, demands a vast number of computations. Consequently, high-speed computation platforms like FPGAs, GPUs, and multi-core systems emerge as appealing options to conduct these simulations within a reasonable timeframe.

## FPGA technology computing platform

Spatially reconfigurable computing technology based on Field Programmable Gate Arrays (FPGAs) has proven successful in addressing challenges across various application domains, including signal and image processing, pattern recognition, real-time guidance and control, deep packet inspection networking, machine learning, cyber-security, and cyber-physical systems [13]. Modern SRAM-based FPGAs are integrated circuits that offer user-configurable capabilities in the field, allowing them to be reprogrammed as needed after fabrication to fulfill specific functions. These FPGAs consist of numerous interconnected small building blocks, forming an on-chip finely-grained-hierarchical switching and routing fabric. These building blocks encompass Adaptive Logic Modules (ALMs), high-speed digital and streaming I/O ports, Digital Signal Processing (DSP), and high-density embedded SRAM Memory blocks, along with elements like Phase-Lock-Loops (PLLs) for internal clock multiplication and skew management [1]. Typically, the internal low-level structure of FPGAs closely aligns with the structure of the application, as the building blocks themselves are not overly complex. In SRAM-based FPGAs, each ALM includes at least one Lookup Table (LUT), selectively feeding into one or more flip flops within the ALM. This design enables efficient implementation of high-speed sequential synchronous designs, with combinational logic segments assigned to the LUTs and associated flip flops serving as the base memory element. Modern FPGAs further enhance performance by leveraging functional and data parallel methods [14–18]. These methods enable simultaneous execution of problem space and/or data space on different FPGA portions, supported by separately addressable on-chip embedded SRAM memory blocks and hierarchical segmentation within the internal interconnect fabric. This parallelism, encompassing both temporal and functional/data aspects, is less sensitive to small data size effects but necessitates explicit user-defined synchronization [19–23].

Energy consumption has long been a critical consideration in mobile computing devices and is increasingly limiting scientific high-performance computing applications [1, 13, 24, 25]. FPGAs have emerged as a solution to reduce overall energy and power consumption for specific applications. This reduction is evident when FPGAs serve as accelerators, offloading complex tasks from the CPU, whether used independently or in conjunction with other platforms [26, 27]. A research paper comparing platforms for random number generator implementation found that FPGAs provide the highest performance per Joule, surpassing CPUs, GPUs, and Massively Parallel Processor Arrays [12, 28]. The FPGA board under investigation is the Intel DE5a-Net board featuring the Arria 10 architecture. This board is equipped with 427,200 Adaptive Logic Modules (ALMs), 1518 DSP blocks, and 2713 RAM blocks. It is imperative that the synthesizable code remains within the confines of the available resources on the board.

Numerous investigations have evaluated different computation platforms concerning computational speed and energy consumption across diverse applications. In the field of image vision applications [29], the Jesson TX2 GPU surpasses the ARM CPU and ZCU102 FPGA in terms of power consumption per frame for straightforward and easily parallelizable vision kernels. However, for more intricate kernels, the FPGA exhibits superior performance, achieving an improvement factor of up to 23 times. Within the field of robotics, a previous investigation [30] delved into accelerating gradients in rigid body dynamics across different computation platforms. The findings underscored the efficacy of employing FPGA and GPU computing platforms, showcasing an enhancement factor of up to 3 when contrasted with the state-of-the-art CPU computation platform. The FPGA computing platform has demonstrated remarkable speed in implementing basic linear algebra subroutines (BLAS) for matrix-to-matrix multiplication [31]. The Xilinx zcu102 FPGA achieved a speedup factor of up to 22 times compared to conventional CPUs and 6 times compared to the utilized GPU platform.

## OpenCL framework

A significant challenge in achieving widespread acceptance of reconfigurable computing lies in expressing intricate designs at a high level of abstraction and efficiently implementing them within FPGA fabric. An effective approach to addressing this challenge has been the introduction of the OpenCL standard. OpenCL, an open standard for encoding applications, is designed for use with CPUs, GPUs, DSPs, and FPGAs. It builds upon the C99 standard and provides application programming interfaces for data and control transfer between a host and one or more accelerator devices [1, 12]. On FPGAs, OpenCL introduces temporal parallelism through its task parallel model, enabling the decomposition of loop-level parallelism into highly pipelined structures within the FPGA fabric. Additionally, OpenCL supports functional/data parallelism on FPGAs through the NDrange model, allowing the replication of computation portions and the use of pipeline structures. Both major FPGA vendors, Xilinx and Intel FPGA, have embraced the OpenCL standard as a high-level synthesis method. The OpenCL programming model comprises two main sections. The first is a host program, typically written in C/C++, executed on a connected CPU to coordinate the activity of accelerators like GPU, FPGA, or DSP. The second is the device code program, written in OpenCL and executable on available devices [15, 16].

In the OpenCL framework, a host connects to one or more devices, each having varied computational structures. Each device consists of one or more compute units, and each compute unit is composed of one or more processing elements. Threads run on compute units, and host programs include kernels—functions executed by one or multiple threads. Kernel implementation and functionality depend on factors such as dependency and shared data between threads [13]. Parameters like the number of compute units, threads per block, vectorization degree, and other parameters are set by the programmer to achieve optimal performance. The Intel FPGA SDK for OpenCL facilitates the implementation of parallel algorithms on FPGA with a high level of hardware abstraction. It generates the FPGA bitmap for execution on the FPGA device. FPGAs typically create pipelining architectures where input data passes through multiple stages. However, the compilation process is time-consuming, ranging from several hours to days, making a just-in-time programming model impractical. Consequently, all FPGA kernels are generated offline.

## Related work

The execution of a PIC simulation typically involves a substantial array of computational functions, and this complexity tends to increase with the growing number of particles. In the past, older simulations relying on less efficient CPUs struggled to handle a large particle count, primarily due to constraints in memory availability [32]. However, modern systems equipped with multiple CPUs provide an avenue for adapting intricate applications to function across multiple machines [33–35]. In the early 90s, a GCPIC concurrent approach was introduced to leverage and distribute PIC simulations across a multi-processor architecture [36]. Nevertheless, applying this algorithm posed numerous challenges, particularly in terms of potential unbalanced loading. The method’s overall performance heavily relied on the most heavily loaded system, presenting a significant drawback. To address the issue of workload balancing, a more recent approach based on dynamic load balancing was introduced [37]. This involved redistributing particles if any processor exceeded the proposed ideal workload by a fixed percentage. However, it’s important to note that data transfer time remains a primary concern despite these advancements. In the context of a 2D-PIC simulation, a strategy was explored wherein the global address was divided among multiple threads using Unified Parallel C (UPC) to achieve enhanced load balancing [38]. This approach successfully contributed to performance improvement, reducing the overall execution time by approximately 25%.

Given the favorable characteristics of GPUs, particularly their numerous processing elements, they present an appealing choice for extensive computations in PIC simulations. Various implementations of PIC simulation exist, each tailored to the differences in GPU architecture. A study conducted by Decyk and Singh presented two conceivable approaches, both designed in accordance with the existing architecture. A notable challenge in these implementations is the need to reorder all particles at every time step, constituting 60% of the total execution time. It is essential for adjacent threads to access adjacent memory locations, and particles updating the same grid point should be stored contiguously [39]. The initial GPU implementation adopted a collision-free algorithm, dividing the grid size into tiles, each managed by one thread. Threads could potentially handle more than one tile, and additional guard cells or grids were introduced to each tile to ensure the independence of all tile calculations. During the calculation of the total charge density at each grid point, each thread processed different particles and wrote to distinct memory locations, enabling parallel execution of all threads. With the continuous evolution of GPU architecture, an alternative implementation involves a collision-solving algorithm specifically designed for Fermi-based GPUs architecture [40]. Fermi GPUs are equipped with larger cache memory and support the use of *atomicadd* on floating point numbers, a desirable feature in PIC implementation. This approach allows for the allocation of multiple threads to each tile, utilizing the *atomicadd* function in charge density calculations. Enlarging the tile size has the benefit of decreasing the number of particles moving between tiles and thereby reducing the time required for particle sorting. Moreover, memory sharing among threads helps minimize the amount of shared memory needed. Nevertheless, there is still a notable overhead due to the essential requirement of reordering particles at every iteration. Efficiency in memory access through the coalescing of data significantly reduces the execution time of the simulation. In a correlated investigation, particles were systematically arranged into a linear array, ensuring that all particles associated with vertex *V* are stored contiguously [41]. Threads in this arrangement perform read/write memory operations in a coalesced manner. This approach necessitates the binning of all particles, wherein they are divided into groups, and each group or bin encompasses a multitude of particles, organized in a sorted fashion. Leveraging CUDA, each bin is processed by one thread block, ensuring that the number of threads aligns with the number of bins. A drawback of this method lies in the constraints of maintaining a uniform bin size. Sewell further implemented the 2D-PIC simulation on the GPU platform [42]. To extract more advantages from the GPU architecture, particles are sorted in both particles-to-grid and grid-to-particle interpolations. The primary objective is to coalesce memory accesses and facilitate the more convenient use of the Single Instruction Multiple Data (SIMD) programming model. As a result, the simulation achieved a remarkable 38-fold acceleration compared to running on a single-core general-purpose processing system [43].

In recent times, numerous studies have delved into the implementation of plasma simulation using Particle-in-Cell (PIC) approaches. These simulations were executed on state-of-the-art massively parallel GPUs from NVIDIA and AMD, resulting in substantial improvements correlated with the number of particles [44]. An optimized PIC code, known as SIMPIC, was developed based on specific hypotheses to implement the proposed PIC on contemporary GPUs. The outcomes of this endeavor underscore the code’s efficiency, showcasing a noteworthy 50% reduction in CPU execution time [45]. Another investigation explored the effective integration of hybrid CPU/GPU platforms for the implementation of large-scale 3D PIC simulations [46]. The framework *PUMIPic* [47] was developed to distribute the overall PIC simulation workload across multiple GPUs arranged in a mesh structure. This framework exhibits the capability to handle simulations with a large number of particles in a more time-efficient manner. WarpX is a developed code library that can be used to study plasma using the PIC approach with the ability to run on multi-core and GPUs computation platforms [48]. It incorporates recent algorithmic enhancements, including boosted frame techniques and refined Maxwell solvers. An additional investigation focused on adapting the PIC code for execution on multi-GPU systems, exemplified by sputniPIC [49]. The findings illustrate the efficacy of this library-based approach in significantly enhancing computation speeds while accommodating large-scale three-dimensional PIC simulations. In a separate study [50], researchers introduced hPIC2, a new library designed for studying plasma interactions via PIC simulations on High-Performance Computing (HPC) systems. The study demonstrates the library’s capability to achieve scalable performance across various computing platforms, particularly evident when simulating large-scale PIC scenarios.

In a prior investigation [12], I utilized the OpenCL framework to enhance the run-time performance and mitigate the overall energy consumption of the proposed 2D-PIC simulation. The primary objective of that research was to alleviate the considerable round-trip latency associated with updating global memory access for grid points, coupled with the prerequisite for completing this operation before proceeding with computations. The outcomes of the study demonstrated an approximate 2.5-fold enhancement in performance and an 8-fold improvement in energy consumption over the lifespan of the simulation, when compared to the reference single-core CPU implementation.

The presented research introduces an innovative architecture designed to minimize the execution time of the most time-consuming phase in PIC simulation. In essence, the contributions of this study can be outlined as follows:

- Introduction of a novel architecture specifically tailored for extensive computations in 2D-PIC simulations, addressing the challenges posed by substantial memory latency and synchronization requirements during the charge accumulation process.
- Optimization of the proposed design to ensure compatibility with various FPGA devices, with the enhancement factor contingent upon the capabilities of the specific FPGA.
- Development of an efficient pipeline architecture capable of executing numerous operations in a single clock cycle, thereby significantly boosting computational efficiency.

## Results and discussion

**Fig 2:**
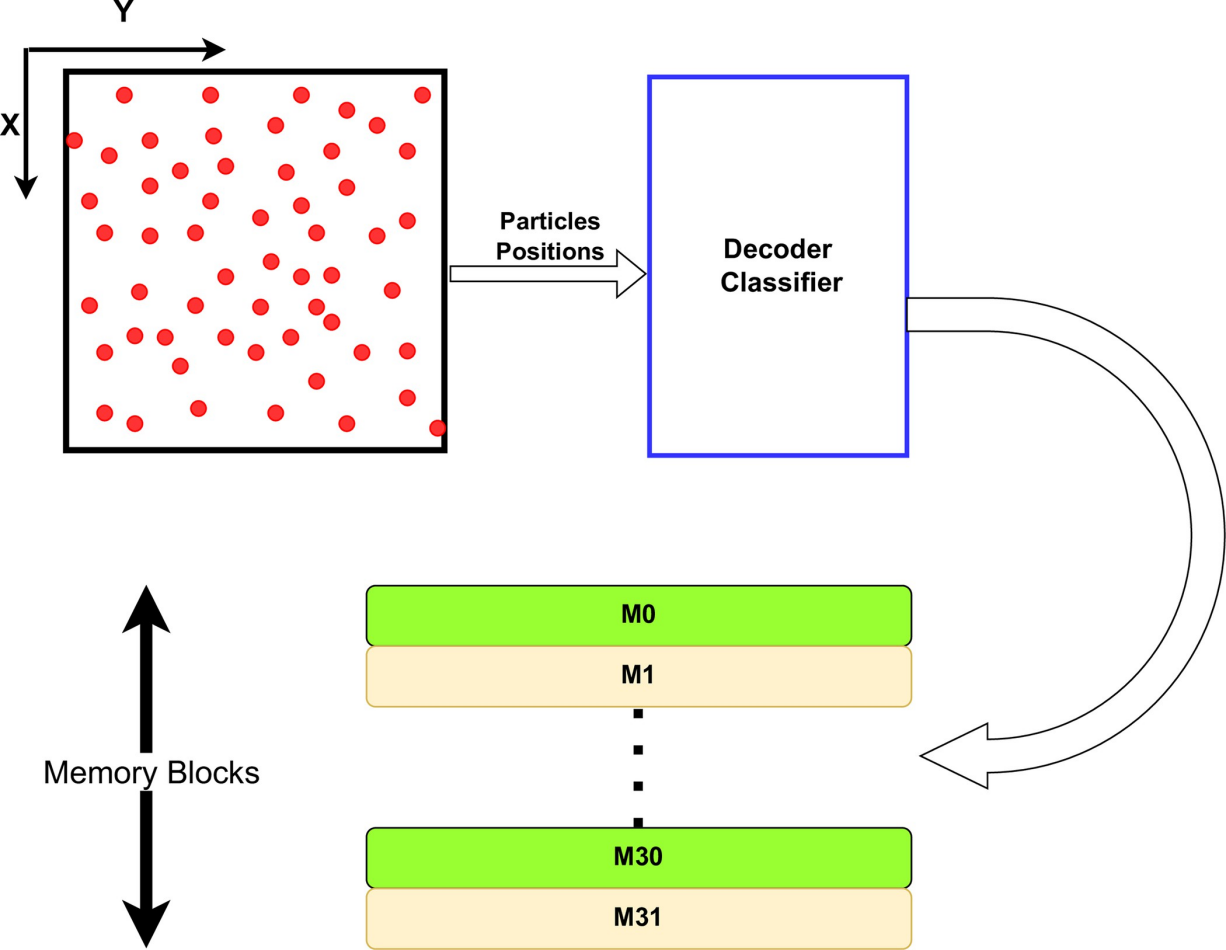
Particles distribution into separate memory buffers.

Managing synchronization is a significant challenge that can emerge in various extensive computational application settings, potentially leading to a notable decrease in overall performance. Specifically addressing the Particles to Grid interpolation, wherein multiple particles concurrently contribute a fraction of the total charge to adjacent grid points, introduces a susceptibility to race conditions. This is due to the potential dependency on the order in which particles contribute their charges, possibly resulting in simultaneous additions to the same grid point. To address this concern, various strategies have been proposed [12, 44, 45]. The proposed strategy introduces a hardware-based solution to tackle the challenge posed by a substantial reduction in performance due to the need for synchronization in performing grid-based computations. Instead of conducting a comprehensive sorting of all particles at each simulation step, which consumes a considerable portion of the overall computation time [44], particles are allocated to distinct memory buffers based on their positions using the suggested hardware architecture, as illustrated in Fig 2. This approach eliminates the necessity for complete particles sorting [1, 2] during each step of the Particle-in-Cell (PIC) simulation, resulting in significant timesaving. Given that PIC simulations often entail thousands of steps to attain a steady-state level [51] or meet specific conditions, this reduction in processing time is particularly beneficial.

**Fig 3:**
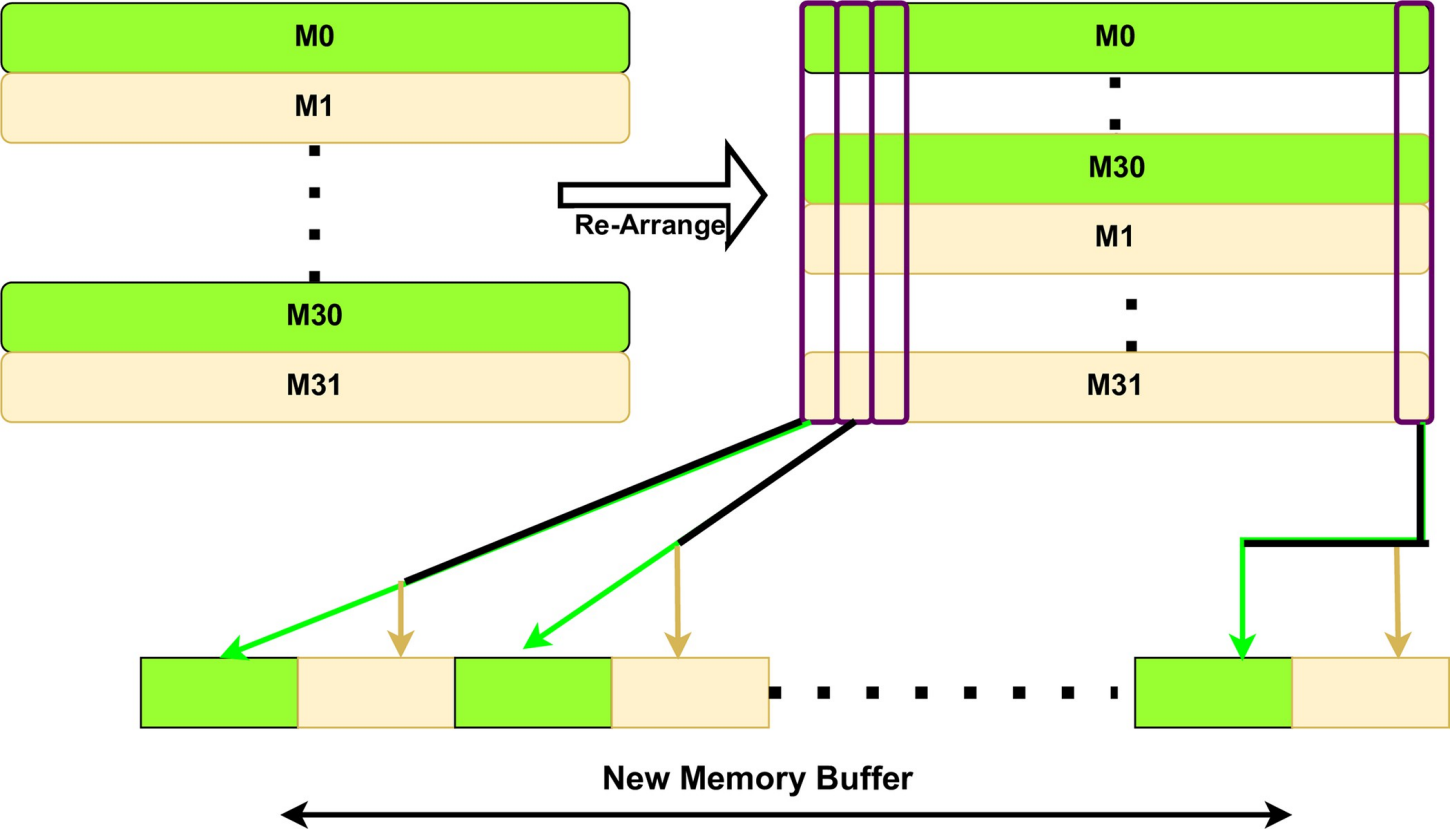
Odd-even memory buffers arrangement.

The execution of the proposed design unfolds in three primary phases. The initial phase involves data preparation and the transfer particles information from the host memory to the global memory of the FPGA device. In the subsequent phase, the data is copied in blocks to customized local memory buffers, followed by the execution of various computations using the proposed functional units. Finally, the intermediate results are written back to the global memory. Particles located within the identical row of the 2D-Grid are allocated to the same shared memory buffer. However, a challenge emerges regarding boundary conditions between consecutive rows of the 2D grid space in this arrangement. To address this concern, a resolution involves reorganizing the memory buffers to prioritize the storage of odd buffers before even buffers, as illustrated in Fig 3. This adjustment tailors the sequence of particle charge interpolation onto the grid. The concept of accelerating computations is derived from establishing a robust pipeline architecture that facilitates the overlapping execution of instructions. The introduction of the odd-even buffer order is implemented to mitigate dependency issues arising between computations involving neighboring grid cells.

**Fig 4:**
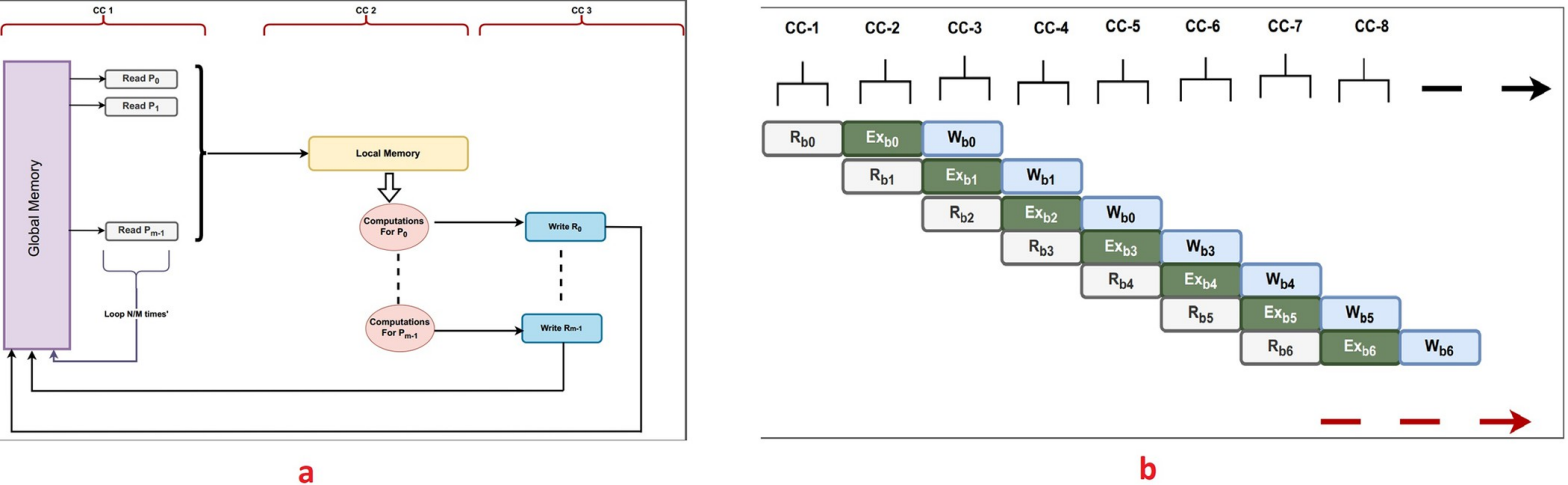
The pipelined execution architecture created using the Intel FPGA synthesizer. (CC: Clock Cycle, R: Read-phase, W: Write-phase, Ex: Execute-phase).

Following the phase of organizing memory, the data undergoes transfer from the host PC to the FPGA global memory. Subsequently, the data is written to the FPGA local memory, where, during each clock cycle, a block of data containing information about non-adjacent particles is copied to the FPGA local memory. In the subsequent clock cycle, a variety of computations, involving additions, subtractions, and comparator hardware circuits, are executed concurrently. This is facilitated by the pre-constructed architecture implemented on the FPGA after an extensive compilations process, resulting in the creation of an efficient hardware design. Ultimately, in the third clock cycle, the corresponding results are written back to another FPGA global memory buffer. The entire process is fully pipelined, aiming to achieve the utmost level of performance optimization, as depicted in Fig 4(A) and 4(B).

To summarize the entire process, initially, particles are uniformly distributed across grid cells, with each particle assigned to a specific buffer based on its x-position. The number of created buffers corresponds to the number of grid rows, exemplified by 64 buffers for a 64x128 grid dimension. The proposed hardware design is intentionally devised to read from nonadjacent even-numbered buffers, followed by nonadjacent odd-numbered buffers in each loop iteration. This design addresses boundary condition issues between adjacent particles, significantly reducing the time required for particle sorting and efficiently allocating each particle to one of the created buffers.

In the subsequent phase, the electrical potential is computed according to the description in Fig 1. However, during the final phase, involving particle movement, particles may transition from their current grid row to one of the adjacent rows, essentially moving between buffers. In this proposed approach, once the new particle location is determined, it can be reassigned to either the same or a different buffer based on its x-location. Buffer contents undergo updates in every time step of the simulation, facilitated by the constructed hardware design, which incorporates all the necessary functional units for implementing the proposed algorithm.

The proposed hardware implementation adopts the Task-parallel model approach [13–16], wherein multiple loop iterations are overlapped during execution. This model is particularly advantageous when there is substantial data sharing, minimizing the significant time associated with data transfer requirements between memory buffers of multiple threads. To address challenges posed by the high cost resulting from potential data dependencies between successive computations, the proposed design incorporates various mechanisms. The utilization of local memory significantly reduces memory access time, and the integration of shift registers increases the distance between dependent computations, enhancing the potential for a robust pipelined architecture.

The Intel FPGA compiler generates multiple files that facilitate dependency identification, pinpoint performance bottlenecks, and provide suggestions for overall design improvement. In the task-parallel mode, the loop unrolling technique is crucial for creating the hardware architecture and increasing the workload per clock cycle. As depicted in Fig 4(A) and 4(B). The proposed design efficiently processes blocks of data instead of individual elements. The innovative concept of establishing multiple and distinct memory banks enables multiple reads/writes from memory buffers. The pragma *ivdep* is employed to signify the potential for reading and writing multiple data in various iterations without encountering dependency issues, a situation the proposed design is primarily designed to avoid.

**Fig 5:**
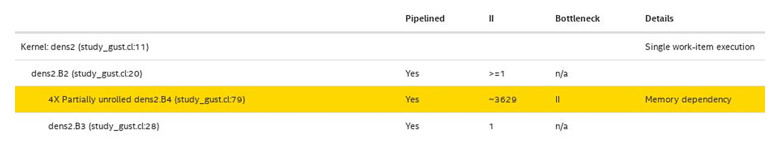
The initial design report with high (II) because of memory dependencies.

**Fig 6:**
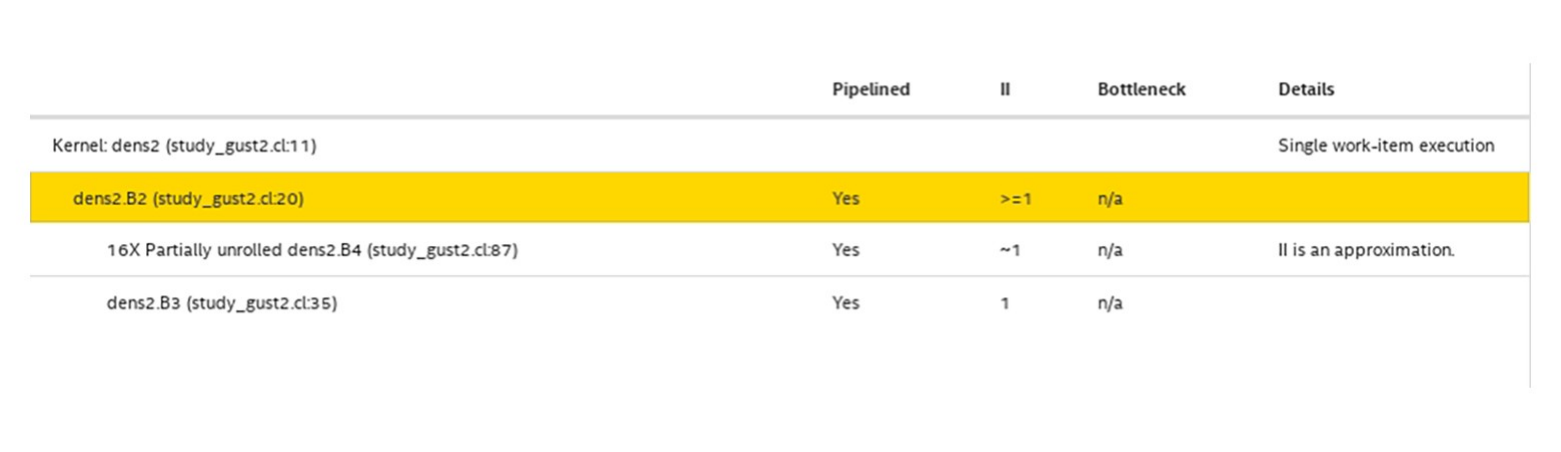
The optimized design report after using several optimizations techniques.

An essential parameter in crafting a robust pipeline design is the initiation interval (*II*), representing the time between two successive operations. Ideally, the *II* is equivalent to one clock cycle. However, data dependencies and extended global memory access times can elevate the *II* to thousands of clock cycles, as illustrated in Fig 5. Nevertheless, the integration of local memory, shift-registers, and the division of global memory into multiple banks narrows the *II*, bringing it closer to the ideal value of one clock cycle, as demonstrated in Fig 6. Loop unrolling also enables the handling of a substantial number of computations by establishing sufficient functional units within the proposed design.

**Fig 7:**
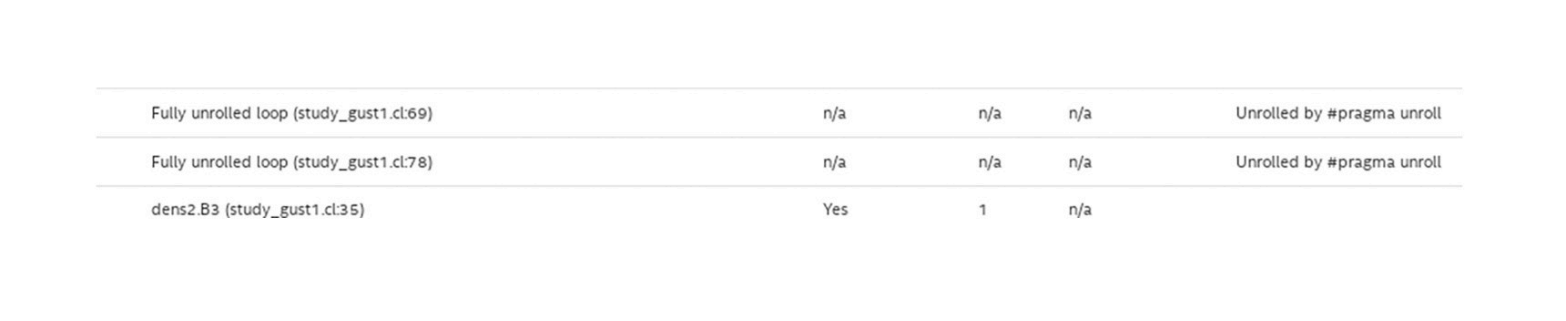
Loop unrolling optimization technique utilized in the proposed implementation.

Loop unrolling technique is employed to fully harness and maximize the FPGA device’s capabilities as shown in Fig 7. Both phases are entirely pipelined, ensuring that several read/write/computation operations are conducted in each phase, as shown also in Fig 4. The proposed design incorporates all necessary functional units for these operations.

Regarding the acceleration achieved by the proposed architecture, its efficacy is predominantly contingent on the clock-cycle time, the workload executed per clock cycle (or the loop-unrolling factor), and the initiation interval. Ideally, an effective pipeline design strives for an *II* close to one clock cycle. However, augmenting the workload per clock cycle may extend the clock-cycle time. Multiple experiments are conducted using the Intel FPGA compiler to optimize the workload per clock cycle and attain a reasonable clock-cycle time.

To address the substantial cost associated with data dependencies, these dependencies are reconfigured to occur between local memory buffers. The utilization of shift registers is then implemented to minimize dependencies and enhance the *II* value. Incorporating multiple memory banks further mitigates dependencies, thereby enhancing the potential to increase the workload per clock cycle. The entire dataset is moved from the host’s global memory to the FPGA device’s global memory, with 32 particle structures being copied every clock cycle. Given a total of 4 million particles, this necessitates approximately 2^19^ clock cycles for completion. Subsequently, the data is transferred to the local memory at a rate of thirty-two particles per clock cycle. The charge interpolation process for all non-overlapping particles takes place in the ensuing clock cycle, followed by the writing of results back to the global memory. This entire procedure is fully pipelined, with charges accumulation for a total of thirty-two particles being processed in each clock cycle. For each particle, there are two absolute value functions, 15 add/subtract functions, and 3 multiplications, summing up to 20 arithmetic operations per particle and a grand total of 640 arithmetic operations in each clock cycle. Despite the relatively large clock-cycle time, where the actual working frequency is 265.7 MHz, when compared to recent CPUs (and being 10 times slower), on the other side a substantial number of operations can be executed. The potential improvement can be up to 60 times, considering each operation, on average, requires one clock cycle.

For a fair comparison, we conduct an approximate estimation of the processing time (in nanoseconds) required for a single particle on the designated FPGA in this study, as well as on similar or different computation platforms employing dissimilar approaches. Although the FPGA chosen here may not represent the most recent and top-tier capabilities in FPGA computation, the proposed methodology can be extended to be implemented on higher FPGA capabilities. In the envisioned approach tailored for the De5 board, an average of eight particles is processed per clock cycle, translating to a requirement of approximately 0.48 ns per particle at the 265 MHz board frequency. To assess the efficiency of our proposed approach, we benchmark our results against a study [52] that introduced a parallel 2D PIC simulation on various GPU platforms (GTX-580, GTX Titan Black, and GTX Titan X). The comparison is based on the total time required to process each particle during a comparable phase of the PIC simulation. The study in question evaluated four algorithms, including a traditional serial approach and a non-sorting algorithm, along with two sorting-based algorithms using different particle-loop and cell-based memory allocation methods. The fourth algorithm leveraged memory coalescing on the GPU for enhanced performance. Examination of Table 1. reveals that the performance achieved by our proposed algorithm closely aligns with the peak performance attained by the GTX Titan GPU (0.41 ns/particle). It’s noteworthy to highlight that in the [52] research study, all GPU acceleration cards employed demonstrated a performance improvement ranging from 80 to 140 times compared to the conventional single-core CPU (Intel Xeon E5620) computation platform.

**Table 1:** The execution times (nano seconds) per particle are measured for four different algorithms [47], (A: first algorithm, B: second algorithm, C: third algorithm: and D: fourth algorithm) using the DE5 FFPGA, GTX 580 GPU, GTX Titan Black and GTX Titan X.

DE5 (This Study)	GTX 580 GPU				GTX Titan Black				GTX Titan X			
	A	B	C	D	A	B	C	D	A	B	C	D
0.47	11.24	13.27	1.72	0.51	5.12	1.24	1.19	0.41	3.38	1.53	0.53	0.42

An additional advantage of utilizing the FPGA card lies in its energy efficiency, with an estimated average dynamic power consumption of 9W for the DE5 board, whereas certain GPU architectures may exceed 100W in power consumption, as per our power analyzer tool estimates. For the purpose of comparing energy consumption, the Thermal Design Power (TDP) serves as a benchmark. The GTX Titan GPU has a TDP of 250W, while the GTX 580 GPU’s TDP is slightly lower at 244W [53]. TDP can provide a proper estimate of the average power consumption under moderate workloads when a processor operates at its base clock [54]. Performing a straightforward calculation in terms of joules consumed per particle shows that in the targeted DE5 FPGA processing, approximately 4.27 nano joules (*nJ)* are required per particle, compared to about 102.5 *nJ* per particle when utilizing the GTX Titan GPU. This implies that the adoption of the proposed FPGA computing platform could significantly improve the power consumption factor by more than 24 times. The approximate joules consumed per particle for the utilized FPGA computing platform and several other GPU computing platforms [52] are summarized in Table 2.

**Table 2:** Approximate energy consumed per particles in *nano Joules* (*nJ*) for various computation platforms and algorithms (A: first algorithm, B: second algorithm, C: third algorithm: and D: fourth algorithm).

DE5 (This Study)	GTX 580 GPU				GTX Titan Black				GTX Titan X			
	A	B	C	D	A	B	C	D	A	B	C	D
4.2	2742.6	3237.9	419.7	127.5	1280.0	310.0	297.5	102.5	845.0	382.5	132.5	105.0

## Conclusion

This research has successfully addressed the formidable computational challenges associated with Particle-in-Cell (PIC) simulations in the investigation of plasma—a crucial state of matter in the universe. The proposed novel implementation approach, focusing specifically on the Particle-to-Interpolation phase, leverages the high-speed capabilities of a Field Programmable Gate Array (FPGA) computation platform. By incorporating various optimization techniques and capitalizing on the flexibility and performance attributes of the Intel FPGA device, our approach significantly diminishes memory access latency, enhancing the efficiency of the PIC simulation. The obtained results underscore the effectiveness of our design, demonstrating the remarkable capability to execute hundreds of functional operations in each clock cycle. This starkly contrasts with the limitations of operations performed on a general-purpose single-core computation platform (CPU). The research study further underscores the significance of employing the FPGA computing platform to enhance the energy consumption factor by reducing it significantly. This groundbreaking research not only introduces a practical solution to the computational bottleneck in PIC simulations but also opens avenues for further advancements in the exploration of plasma characteristics. The optimized FPGA-based approach showcased in this study holds great promise for accelerating research in plasma physics and related fields, providing a valuable contribution to the scientific community’s understanding of complex plasma phenomena.

## Supporting information

S1 Fig (JPG)

S2 Fig (JPG)
