# Correction: Accelerating electrostatic particle-in-cell simulation: A novel FPGA-based approach for efficient plasma investigations

**DOI:** 10.1371/journal.pone.0316005

**Published:** 2024-12-13

**Authors:** Abedalmuhdi Almomany, Muhammed Sutcu, Babul Salam K. S. M. Kader Ibrahim

The affiliation for the first and second author is incorrect. Abedalmuhdi Almomany is not affiliated with #2 but with #3: 3 Department of Computer Engineering, Hijjawi Faculty for Engineering Technology, Yarmouk University, Irbid, Jordan. Muhammed Sutcu is not affiliated with #3 but with #2: Department of Engineering Management, Gulf University for Science & Technology, Hawally, Kuwait.
