# Supplementary figures and images for: Accelerating electrostatic particle-in-cell simulation: A novel FPGA-based approach for efficient plasma investigations
Source: PLoS One. 2024 Jun 3;19(6):e0302578. doi: 10.1371/journal.pone.0302578 (PMC11146701; doi:10.1371/journal.pone.0302578)

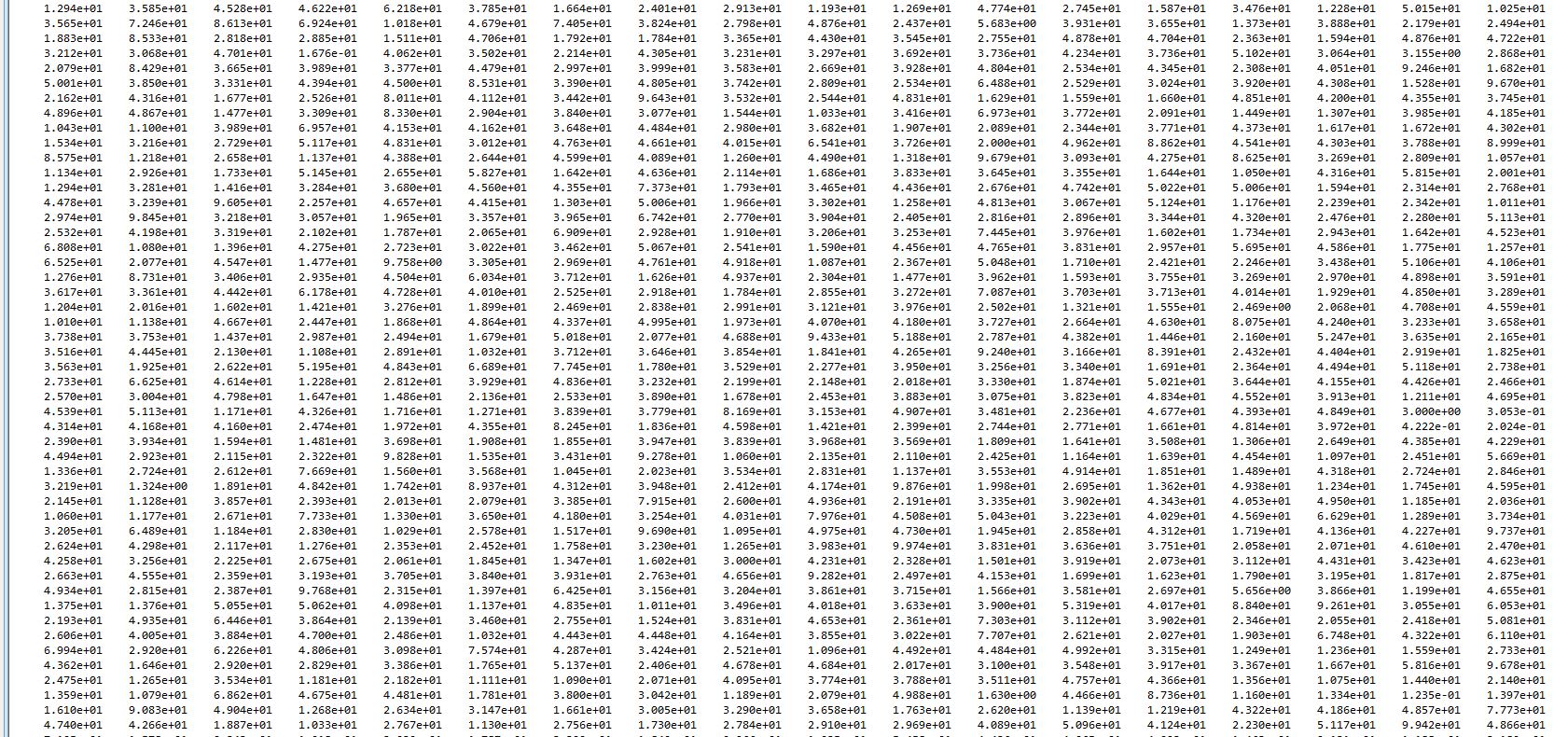

Supplement: S1 Fig — (JPG) [file pone.0302578.s001.JPG]

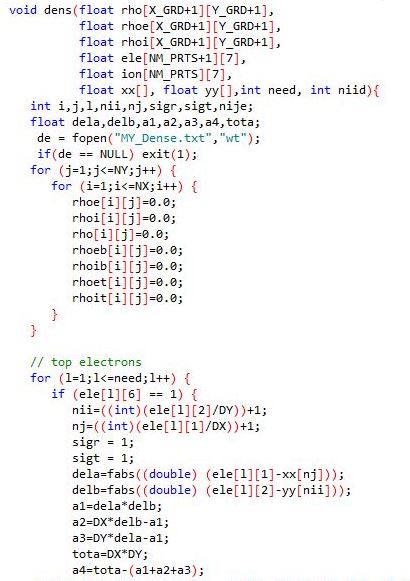

Supplement: S2 Fig — (JPG) [file pone.0302578.s002.JPG]
